# Affluence and Private Health Insurance Influence Treatment and Survival in Non-Hodgkin’s Lymphoma

**DOI:** 10.1371/journal.pone.0168684

**Published:** 2016-12-19

**Authors:** Harry Comber, Marianna De Camargo Cancela, Trutz Haase, Howard Johnson, Linda Sharp, Jonathan Pratschke

**Affiliations:** 1 National Cancer Registry, Cork, Ireland; 2 Brazilian National Cancer Institute, Epidemiology Division, Rio de Janeiro, Brazil; 3 Social and Economic Consultant, Dublin, Ireland; 4 Health & Wellbeing Directorate Health Intelligence Unit, Health Service Executive, Dublin, Ireland; 5 Institute of Health & Society, Newcastle University, Newcastle upon Tyne, United Kingdom; 6 Department of Economics and Statistics, University of Salerno, Salerno, Italy; University of Cincinnati College of Medicine, UNITED STATES

## Abstract

**Background:**

The aim of this study was to investigate inequalities in survival for non-Hodgkin’s lymphoma (NHL), distinguishing between direct and indirect effects of patient, social and process-of-care factors.

**Methods:**

All cases of NHL diagnosed in Ireland in 2004–2008 were included. Variables describing patient, cancer, stage and process of care were included in a discrete-time model of survival using Structural Equation Modelling software.

**Results:**

Emergency admissions were more common in patients with co-morbid conditions or with more aggressive cancers, and less frequent for patients from more affluent areas. Aggressive morphology, female sex, emergency admission, increasing age, comorbidity, treatment in a high caseload hospital and late stage were associated with increased hazard of mortality. Private patients had a reduced hazard of mortality, mediated by systemic therapy, admission to high caseload hospitals and fewer emergency admissions.

**Discussion:**

The higher rate of emergency presentation, and consequent poorer survival, of uninsured patients, suggests they face barriers to early presentation. Social, educational and cultural factors may also discourage disadvantaged patients from consulting with early symptoms of NHL. Non-insured patients, who present later and have more emergency admissions would benefit from better access to diagnostic services. Older patients remain disadvantaged by sub-optimal treatment, treatment in non-specialist centres and emergency admission.

## Introduction

International studies have shown a rapid increase in incidence of non-Hodgkin’s lymphoma (NHL) [1–5] in many countries from the early 1970s onwards and significant improvements in survival from the mid 1990s [2,3,6–9]. Survival differences persist between countries [6], although they are smaller than for many solid tumours. Differences in NHL survival by sex, ethnicity and socio-economic status are also observed within countries [10–16]. While some differences in survival between groups may be due to differences in tumour biology [17], most are probably attributable to differences between groups in underlying health and in the use of, access to, and experience of, health services. Poorer outcomes may be related to age, sex, marital status, ethnicity, relative poverty, geographical isolation or social isolation and may be exacerbated by conscious or unconscious discrimination in health service access, operation or configuration [18–22].

Investigation of the impact of these factors on survival should distinguish between direct and indirect effects of personal attributes and differential access to services. Factors like comorbidity or age may have direct effects on survival or may operate indirectly via associations with, for example, treatment. Deprived patients may have financial, cultural or transport difficulties in accessing diagnostic or treatment services and may suffer discriminatory treatment by health providers [23]. Conventional survival modelling can control for these factors—insofar as they are measurable—but cannot provide information on the complex pathways through which deprivation influences survival, or on the relative magnitudes of these indirect effects; this information is only available if direct and indirect influences are distinguished. Without this information, action to mitigate deprivation may be targeted inappropriately.

The aim of this study was to investigate inequalities in survival for patients diagnosed with NHL, distinguishing between direct and indirect effects of patient, social and health service-related factors. The methodology used in this paper is particularly suited to the study of survival in NHL, which is close to that of all cancers combined [24] and is strongly dependent on age, stage and treatment. We have attempted to overcome the limitations of current models by using an alternative analytical approach. This involves extending Structural Equation Modelling to include survival outcomes, via an innovative discrete-time specification of the hazard. This has allowed us to explore the complex relationships between variables and to distinguish between direct and indirect effects on survival.

## Methods

All cases of NHL registered by the Irish National Cancer Registry as incident during the years 2004–2008 were included. Patients registered with a subsequent invasive cancer (other than non-melanoma skin cancer) between 1/1/2004 and 31/12/2011 were excluded as it was not possible to determine which cancer would be the cause of death. Completeness of registration of cancer at the Registry has been estimated to be at least 97–98% [25].

Registry data were linked, using probabilistic matching on name, address and date of birth, to public hospital discharge data from the Hospital Inpatient Enquiry (HIPE) for all patients admitted to public hospitals [26]. 86% of cases could be linked in this way to at least one HIPE record. The public/private status of patients (i.e. if they paid directly, or through health insurance, for some or all of their treatment or diagnostic procedures) is recorded in the HIPE data for patients admitted to public hospitals. Patients admitted to private hospitals only did not have a HIPE record and were classified as “private”. Patients who attended both public and private hospitals for their cancer treatment were assigned one of these two categories on the basis of their longest admission.

The type of initial admission (scheduled or emergency) was determined from HIPE data, as was co-morbidity. HIPE data includes information on up to 19 co-morbid conditions for each discharge and this information was used to assign a Charlson comorbidity score [27], excluding NHL, for each patient. No admission type or Charlson score could be assigned to patients (380, 13.6%) who were never admitted to a public hospital, and therefore had no HIPE record; these patients were assigned a Charlson score of 0 and defined as scheduled admissions.

Information on patient age, address, sex and marital status, tumour stage (Ann Arbor) and grade (indolent or aggressive, based on histological type) at diagnosis was provided by the Registry. Stage information was missing in 14.7% of cases and was assigned using the EM algorithm. Lymphomas were classified as “aggressive” if the ICDO3 morphology [28] was described as any of the following: mantle cell lymphoma; malignant lymphoma (mixed small and large cell), diffuse; malignant lymphoma (large B-cell), diffuse; malignant lymphoma (large B-cell), diffuse, immunoblastic, NOS; Burkitt lymphoma, NOS; follicular lymphoma, grade 3; mature T-cell lymphoma, NOS; angioimmunoblastic T-cell lymphoma; anaplastic large cell lymphoma, T-cell and Null cell type; hepatosplenic gamma-delta cell lymphoma; intestinal T-cell lymphoma; NK/T-cell lymphoma, nasal and nasal-type; precursor cell lymphoblastic lymphoma, NOS; or precursor T-cell lymphoblastic lymphoma. 913 cases were coded as 9590/3 and 9591/3 (NHL or malignant lymphoma, not otherwise specified) and were their grade was classified as “unknown”; the remainder of histological types were classified as “indolent”. A Pobal (HP) area-based deprivation score was assigned to each case, based on the census small area of residence (average population ~230 persons) at the time of diagnosis [29]. This score was unknown in 8.0% of cases, and was assigned using the EM algorithm. Population density of the area of residence was obtained from the 2006 census of population (www.cso.ie) [30] and used as a measure of urban/rural residence. Addresses were also assigned to one of four Health Service Executive (HSE) Regions, two of which include Dublin and two which cover the South and West of the country. These regions are largely self-sufficient with regard to adult cancer services.

Active cancer-directed treatment was defined as systemic therapy, radiotherapy or surgery, where the primary aim was to destroy, or reduce the extent of, the lymphoma. Treatment was classified as “any systemic therapy” (chemotherapy, immunotherapy, targeted therapy) or “other” (radiotherapy, surgery or no active tumour-directed treatment). The hospital of main treatment was determined for each patient from National Cancer Registry data. In most cases, this was the hospital in which the patient had systemic therapy; for patients who did not have systemic therapy, the main hospital was that of radiotherapy, any other tumour-directed treatment or diagnosis. Caseload for the main hospital was calculated as the annual average number of patients with NHL admitted (whether or not they had active tumour-directed treatment) during the study period. The highest quartile of caseload (four hospitals treating >30 patients/year) was defined as “high caseload”.

Registry data were linked to official death certificate data from the Central Statistics Office. Deaths were classified as either due to NHL, or to other causes, using an algorithm developed by the Scottish Cancer Registry [31]. The censoring date was 31/12/2012.

**Fig 1:**
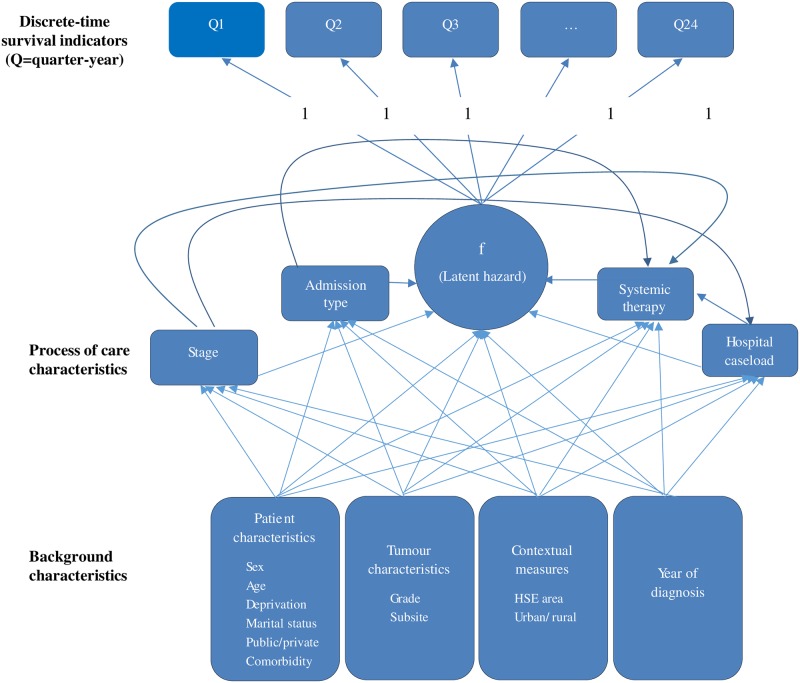
Model structure.

Survival was modelled using a discrete-time survival model using only time-invariant covariates with constant hazards. The methods have been described in detail previously [32]. All models were estimated using version 5.21 of the software package MPlus using the MLR estimator [33]. This allows the specification of a complex model including direct and indirect effects. The structure of the model is illustrated in Fig 1, using the typical conventions for path diagrams. These are modelled by assuming that there is a normally-distributed latent response variable underlying each ordinal dependent variable. The discrete values of the categorical variable coincide with thresholds on the scale of this latent variable or liability factor. The first component of the model comprises 24 dichotomous indicators containing quarterly survival data, covering six years from the moment of diagnosis, and a latent hazard factor which captures the propensity of death during each interval. The second component consists of the remaining variables, grouped into eight blocks: patient characteristics, tumour characteristics, contextual measures, year of diagnosis, stage of disease, type of admission, systemic therapy and hospital caseload. The last four of these are considered as describing the process of care, and the others the background characteristics of the patient and cancer. The model assumes that the background characteristics influence the stage of the cancer at the moment of diagnosis (i.e. early/late diagnosis), whilst treatment optimality is influenced, once again, by background characteristics, the stage of the disease, the caseload of the hospital where treatment is received and the route by which the patient entered the hospital. Caseload and entry route are also regressed on the background characteristics, with a view to exploring their role as mediating factors. Finally, the survival prospects of the patient depend on the combined effect of all of these influences.

In order to simplify interpretation of the indirect effects, we report the results of a model which specifies classical linear regression equations for all dependent variables, regardless of their measurement scale (with the exception of the dichotomous survival outcome, which is modelled using a standard logit specification). A sensitivity analysis was carried out and confirms that the sign and *p*-values for model coefficients are not unduly influenced by this specification.

## Results

### Study population

2,793 cases of NHL, incident in 2004–2008, were included in the analysis (Table 1). 54% of patients were male and 63% were aged under 70. The majority (58%) were married and two-thirds (64%) attended hospital solely, or predominantly, as public patients. 41% of cancers were at Ann Arbor stage I or II at diagnosis, and 41% were classified as having aggressive morphology. 79% of patients had no recorded comorbid conditions and 19% were admitted as an emergency. Just over two-thirds (68%) had systemic therapy, either alone or in combination; 23% had no active cancer-directed treatment.

**Table 1:** Patient, cancer and treatment characteristics (N = 2,793).

*Variable*	*Value*	*Number of cases (%)*
year of incidence	2004	542 (19.4%)
	2005	511 (18.3%)
	2006	576 (20.6%)
	2007	571 (20.4%)
	2008	593 (21.2%)
age at diagnosis	<60	1083 (38.8%)
	60–69	677 (24.2%)
	70–79	661 (23.7%)
	80+	372 (13.3%)
sex	male	1500 (53.7%)
	female	1293 (46.3%)
marital status	married	1613 (57.8%)
	unmarried	1180 (42.2%)
main category of care (private or public)	public	1776 (63.6%)
	private	1017 (36.4%)
HSE area	Dublin Mid Leinster	778 (27.9%)
	Dublin North East	568 (20.3%)
	South	732 (26.2%)
	West	701 (25.1%)
urban/rural residence	urban	986 (35.3%)
	intermediate	598 (21.4%)
	rural	943 (33.8%)
	unknown	266 (9.5%)
grade of tumour	indolent	734 (26.3%)
	aggressive	1146 (41.0%)
	unknown	913 (32.7%)
comorbidities	none/unknown	2210 (79.1%)
	1 or more	583 (20.9%)
tumour stage	Stage I	704 (25.2%)
	Stage II	432 (15.5%)
	Stage III	622 (22.3%)
	Stage IV	625 (22.4%)
	unknown	410 (14.7%)
first hospital admission type	planned	2265 (81.1%)
	emergency	528 (18.9%)
main treatment in high-caseload hospital	no	2098 (75.1%)
	yes	695 (24.9%)
systemic therapy	no	884 (31.7%)
	yes	1909 (68.3%)

### Associations between background characteristics and process variables

The association of background patient and cancer characteristics with four process-of-care variables—stage, emergency admission, hospital caseload and systemic therapy—is shown in Table 2.

**Table 2:** Regression coefficients (beta) of optimum treatment, caseload, tumour stage and first hospital admission type on background variables. Significant results (p<0.05) are shown in bold.

		stage III/IV	emergency admission	caseload>30 cases/year	systemic therapy
**Background variables**					
Sex	male	0.00 (ref))	0.00 (reference class)	0.00 (reference class)	0.00 (reference class)
	female	**0.53 (0.22, 0.84)**	0.00 (-0.12, 0.12)	-0.06 (-0.20, 0.08)	0.00 (-0.12, 0.12)
age	each 10 years	**0.09 (0.05, 0.13)**	0.00 (-0.02, 0.02)	**-0.01 (-0.03, 0.01)**	**-0.06 (-0.08, -0.04)**
older men	all females; males aged <70	0.00 (reference class)	0.00 (reference class)	0.00 (reference class)	0.00 (reference class)
	males 70+	**-0.07 (-0.13, -0.01)**	0.00 (-0.02, 0.02)	0.01 (-0.01, 0.03)	0.00 (-0.02, 0.02)
affluence	per unit score	**-0.33 (-0.60, -0.06)**	**-0.19 (-0.29, -0.09)**	0.07 (-0.05, 0.19)	0.04 (-0.08, 0.16)
marital status	unmarried	0.00 (reference class)	0.00 (reference class)	0.00 (reference class)	0.00 (reference class)
	married	0.02 (-0.06, 0.10)	-0.01 (-0.05, 0.03)	0.00 (-0.04, 0.04)	**0.10 (0.06, 0.14)**
public/private status	public	0.00 (reference class)	0.00 (reference class)	0.00 (reference class)	0.00 (reference class)
	private	-0.06 (-0.16, 0.04)	**-0.03 (-0.07, 0.01)**	**-0.08 (-0.12, -0.04)**	**-0.05 (-0.09, -0.01)**
comorbidities	none	0.00 (reference class)	0.00 (reference class)	0.00 (reference class)	0.00 (reference class)
	one or more	**0.15 (0.05, 0.25)**	**0.10 (0.06, 0.14)**	**0.08 (0.04, 0.12)**	0.00 (-0.04, 0.04)
area of residence	Dublin Mid Leinster	0.00 (reference class)	0.00 (reference class)	0.00 (reference class)	0.00 (reference class)
	Dublin North east	0.00 (-0.12, 0.12)	**-0.04 (-0.08, 0.00)**	0.03 (-0.01, 0.07)	0.03 (-0.03, 0.09)
	South	-0.08 (-0.20, 0.04)	**-0.10 (-0.14, -0.06)**	0.02 (-0.02, 0.06)	**0.05 (0.01, 0.09)**
	West	0.01 (-0.11, 0.13)	0.00 (-0.04, 0.04)	**0.10 (0.06, 0.14)**	**0.07 (0.01, 0.13)**
urban/rural residence	urban	0.00 (reference class)	0.00 (reference class)	0.00 (reference class)	0.00 (reference class)
	intermediate	0.07 (-0.05, 0.19)	0.02 (-0.02, 0.06)	**-0.07 (-0.11, -0.03)**	-0.01 (-0.05, 0.03)
	rural	0.03 (-0.09, 0.15)	0.03 (-0.01, 0.07)	**-0.13 (-0.17, -0.09)**	-0.01 (-0.05, 0.03)
	unknown	-0.03 (-0.19, 0.13)	0.05 (-0.01, 0.11)	**-0.11 (-0.17, -0.05)**	-0.02 (-0.08, 0.04)
tumour grade	low	0.00 (reference class)	0.00 (reference class)	0.00 (reference class)	0.00 (reference class)
	high	**-0.14 (-0.24, -0.04)**	**0.10 (0.06, 0.14)**	0.04 (0.00, 0.08)	**0.22 (0.18, 0.26)**
year of diagnosis	per year	**0.05 (0.01, 0.09)**	**-0.02 (-0.04, 0.00)**	-0.01 (-0.03, 0.01)	0.00 (-0.02, 0.02)
**Process variables**					
stage	I/II	-	0.00 (reference class)	0.00 (reference class)	0.00 (reference class)
	III/IV	-	**0.04 (0.02, 0.06)**	0.01 (-0.01, 0.03)	0.01 (-0.01, 0.03)
emergency admission	no	0.00 (reference class)	-	0.00 (reference class)	0.00 (reference class)-
	yes	**0.04 (0.02, 0.06)**	-	0.01 (-0.01, 0.03)	**-0.70 (-0.74, -0.66)**
hospital caseload	<30 cases/year	0.00 (reference class)	0.00 (reference class)	-	0.00 (reference class)
	30 cases+ per year	0.01 (-0.01, 0.03)	0.01 (-0.01, 0.03)	-	-0.01 (-0.05, 0.03)
systemic therapy	no	0.00 (reference class)	0.00 (reference class)-	0.00 (reference class)	
	yes	0.01 (-0.01, 0.03)	**-0.70 (-0.74, -0.66)**	-0.01 (-0.05, 0.03)	

Presentation at late (III/IV) stage increased significantly over the study period. Late stage was more frequent for female patients and older patients, and those with one or more comorbidities; it was less frequent in those living in more affluent areas and for more aggressive lymphomas. Emergency admissions with lymphoma decreased significantly during the study period and were more common in patients with one or more co-morbid conditions or with more aggressive cancers, and less frequent for patients from more affluent areas, or living in the Dublin North-East and South HSE regions.

Treatment in high caseload hospitals was more common for patients with one or more comorbidities, and for those living in the HSE West region; it was less common for private patients, for those living outside urban areas and for those presenting as an emergency. Systemic therapy was more frequent for married patients, those living in the South and West HSE regions and for patients with more aggressive or late stage lymphomas. Systemic therapy was less common for private patients and for those admitted to hospital as emergency cases.

### Effects on survival

Tables 3 and 4 show the direct and indirect effects, respectively, of both background and process-of-care variables on the hazard, expressed as logit coefficients. Factors associated with an increased hazard of mortality were, in decreasing order of effect size, aggressive morphology, female sex, emergency admission, increasing age, comorbidity, residence in the South HSE region, treatment in a high caseload hospital and late stage. The hazard of mortality was significantly reduced by systemic therapy and private patient status, after controlling for the other variables. In terms of indirect effects, married patients had a reduced hazard of mortality, which was mediated by a higher rate of systemic therapy. Affluence also reduced the hazard indirectly, by reducing late stage and emergency admission, and private patients had a reduced hazard mediated through systemic therapy, admission to high caseload hospitals and fewer emergency admissions. The indirect effects were statistically significant for marital status and affluence, but for private patients the negative effect of lower rates of systemic therapy largely cancelled out the positive impact of admission to higher caseload hospitals and reduced emergency admissions.

**Table 3:** Direct effects (beta) of patient, cancer and treatment characteristics on the hazard.

Variable	Value	Hazard coefficient (95% confidence intervals)
Background variables		
sex	male	0 (reference)
	female	**1.00 (0.20, 1.80)**
age	each 10 years	**0.44 (0.34, 0.54)**
older men (age*sex interaction)	all females; males aged <70	0 (reference)
	males 70+	**-0.12 (-0.24, 0.00)**
affluence	per unit score	-0.02 (-0.41, 0.37)
marital status	unmarried	0 (reference)
	married	-0.04 (-0.20, 0.12)
public/private status	public	0 (reference)
	private	**-0.19 (-0.35, -0.03)**
comorbidities	none	0 (reference)
	one or more	**0.41 (0.23, 0.59)**
area of residence	Dublin Mid Leinster	0 (reference)
	Dublin North east	0.01 (-0.21, 0.23)
	South	**0.35 (0.15, 0.55)**
	West	-0.07 (-0.29, 0.15)
area population density	urban	0 (reference)
	intermediate	0.02 (-0.20, 0.24)
	rural	0.10 (-0.10, 0.30)
	unknown	0.14 (-0.11, 0.39)
tumour grade	indolent	0 (reference)
	aggressive	**1.02 (0.80, 1.24)**
year of diagnosis	per year	-0.04 (-0.10, 0.02)
Process variables		
stage at diagnosis	I/II	0 (reference)
	III/IV	**0.24 (0.18, 0.30)**
emergency admission	No	0 (reference)
	Yes	**0.68 (0.50, 0.86)**
hospital caseload	<30 cases/year	0 (reference)
	30 cases+ per year	**0.21 (0.05, 0.37)**
systemic therapy	No	0 (reference)
	Yes	**-0.53 (-0.69, -0.37)**

**Table 4:** Indirect effects (beta, logit coefficient) of patient, cancer and treatment characteristics on hazard.

	Hazard		
	married (.v. unmarried)	affluence (per unit score)	private patient (.v. public)
systemic therapy	**-0.05 (-0.08, -0.03)**	-0.02 (-0.08, 0.04)	**0.03 (0.01, 0.05)**
high caseload	0.00 (-0.01, 0.01)	0.01 (-0.01, 0.04)	**-0.02 (-0.03, 0.00)**
late stage	0.00 (-0.02, 0.02)	**-0.08 (-0.14, -0.01)**	-0.02 (-0.04, 0.01)
emergency admission	-0.01 (-0.03, 0.01)	**-0.13 (-0.20, -0.05)**	**-0.02 (-0.04, 0.00)**
high caseload→ systemic therapy	<0.01	0.00 (0.00, 0.00)	<0.01
late stage→ systemic therapy	<0.01	**0.04 (0.00, 0.08)**	<0.01
late stage→high caseload	<0.01	<0.01	<0.01
late stage→emergency admission	<0.01	**-0.01 (-0.02, 0.00)**	<0.01
emergency admission→ systemic therapy	<0.01	**-0.01 (-0.01, 0.00)**	<0.01
emergency admission→high caseload	<0.01	**0.01 (0.00, 0.01)**	<0.01
late stage→high caseload→ systemic therapy	<0.01	<0.01	<0.01
late stage→emergency admission→ systemic therapy	<0.01	<0.01	<0.01
late stage→emergency admission→high caseload	<0.01	<0.01	<0.01
late stage→emergency admission→high caseload→ systemic therapy	<0.01	<0.01	<0.01
All indirect effects	**-0.06 (-0.09, -0.02)**	**-0.18 (-0.28, -0.07)**	-0.02 (-0.06, 0.01)
Direct effect*	-0.04 (-0.20, 0.12)	0.08 (-0.41, 0.57)	**-0.19 (-0.35, -0.03)**
Total effect	-0.10 (-0.25, 0.06)	-0.10 (-0.60, 0.39)	**-0.21 (-0.37, -0.05)**

Each cell shows the indirect effects of the column variable on hazard, as mediated through the row variable e.g. the change in hazard of being married, due to receipt of systemic therapy, is -0.05 (-0.08, -0.03).

Statistically significant hazards are shown in bold.

* as shown in Table 2

## Discussion

This study illustrates the complexity of the pathways leading to better or worse survival prospects for patients with NHL. Many of the determinants of survival identified in this study are well-established in the literature—age, socio-economic characteristics, stage, cancer aggressiveness and comorbidity [10,12,13,16,34]. Living in a more affluent area had no significant direct effect on the hazard of mortality, but nevertheless exerted a significant indirect effect, mediated by a lower probability of late stage and emergency presentation. Private patients also had a lower hazard of mortality, but for different reasons [35]—a direct reduction of the hazard, probably due to lower levels of unrecorded comorbidity [36,37] and indirect effects due to lower rates of emergency admission and, somewhat unexpectedly, the fact that admission to higher caseload hospitals was associated with a higher hazard. Private patients were less likely to have systemic therapy, which led to an increase in the hazard of mortality.

The negative impact of higher hospital caseload on survival may be due to differences in case-mix not captured by this analysis. We could find only one study of the effects of caseload on NHL outcome [38]; this looked at physician caseload and found no effect. Patients admitted to high caseload hospitals had a higher level of comorbidity, which was controlled for in this analysis, but it is probable that higher caseload hospitals simply see more complex patients. This complexity may not be fully captured by the comorbidity data available in the HIPE system and the difference in comorbidity between patients in high and low caseload hospitals may be greater than measured here. It should also be noted that only a small number of hospitals were classified as “high caseload” and the finding may be chance.

Although poorer survival and lower treatment rates are often accepted as inevitable results of ageing and increasing co-morbidity, systemic therapy and radiotherapy for NHL are generally well tolerated by older patients [39,40]. We found that systemic therapy was associated with significantly better survival. Older patients in this study were significantly less likely to have systemic therapy, had poorer survival and were less likely to be admitted to a high caseload hospital. Under-treatment of older patients, not explained by co-morbidity, has been frequently reported [41–43] and may reflect attitudes and beliefs both within and outside the health services. Although being married had no independent direct effect on hazard, it had a significant indirect effect, as married patients were more likely to have systemic therapy. Associations between marital status and survival are increasingly reported [17,44–47], but this analysis suggests that these are not direct effects but rather because married patients are more likely to receive optimal treatment [48,49], possibly due to intervention by family members [50].

The reasons for poorer outcomes in NHL patients who present as an emergency are unclear. The proportion of emergency admissions in this study was lower (19% as opposed to 27%) than in a recent large English study [51], which also showed an increase in emergency admission with increasing deprivation. However, we had no information on type of admission for 13.6% of patients, so 19% may be an under-estimate. Unlike the English study, we found no sex difference, after adjustment, in the proportion of emergency admissions. Initial management of elective and emergency lymphomas was similar (data not shown), but there may have been complexities in emergency management not captured here. Significantly more comorbidity was recorded for emergency admissions, but this (as well as stage) was controlled for in the analysis. Not all relevant prognostic data can be routinely recorded by cancer registries [37], and more in-depth analysis (likely involving primary data collection) will be needed to better understand the effects of emergency presentation.

Patients from more affluent areas, and those treated privately (i.e. with private health insurance), had fewer emergency admissions, and consequently better survival, although private health insurance was not related to stage at diagnosis. The higher rate of emergency presentation by uninsured patients suggests that they face greater barriers to earlier presentation. This is unlikely to be at primary care level, as all patients, with the exception of those with the lowest income, must pay a fee of about €60 per visit (and this is generally not covered by health insurance). Hospital waiting times for public patients are lengthy in Ireland [52, 53]. The Irish health system is a very complex mixed public-private system. All residents have access to low-cost secondary care (including all phases of cancer treatment) with small co-payments, but many purchase private health insurance and health care, which tend to coincide. About 44% of the population held private health insurance at the time of the study [54]; this generally covers inpatient care in private hospitals, and inpatient care as a private patient of a consultant in a public hospital.

Emergency presentation may occur if the patient has severe systemic symptoms or obstructive symptoms due to extranodal disease. For the majority of patients, however, symptoms are insidious and non-specific, and an enlarged lymph node or other mass is usually the first diagnostic sign [55]. As with breast [56, 57] and testicular [58–60] cancer, socio-economic factors may lead to cultural and educational differences causing delay before presenting with a lump. These delays may contribute to late or emergency presentation. Patients may also present to different specialties, either medical or surgical, which has the potential to generate further delays in diagnosis and treatment.

Overall, we observed significant disparities in stage at diagnosis and treatment of NHL due to socio-economic status, whether based on area of residence or health insurance. The effect of measures at individual level differed from those measured at the area level, as the latter may have been mediated by geographical factors such as proximity to hospitals and local availability of general practitioners. Clearly, measures constructed at the area and individual levels provide different insights and both should be used whenever possible.

The impact of deprivation on survival is well known, but the mechanisms through which it exerts its effects have been difficult to elucidate. This is at least partly due to the limitations of conventional regression and survival models. The limitations of routinely collected registry data, such as we use here, to examine the causes of socio-economic variation in survival have also been well described [37]. Patients in lower socio-economic groups, or living in “deprived” areas, have been shown to present later [16,17], to have higher levels of co-morbidity [12,17] and to be less likely to have radiotherapy [12,61]. These factors are clearly inter-related [19] but few studies have attempted to unravel the complex configurations of risk factors that appear to be at work. This can be adequately described only by using a more flexible class of statistical model, such as that used in this analysis. A key finding of this study is that deprivation may influence outcomes in multiple ways, each of which needs an appropriate and targeted form of intervention. These models can not only quantify the importance of different pathways, but can also illustrate areas where more information would lead to better understanding.

As already mentioned, a key limitation of this type of study is its retrospective nature and dependence on routinely collected data. The factors determining a patient’s decision to seek help for symptoms, the doctor’s decision to investigate and shared decisions on treatment are complex, and need to be explored. We were also limited by significant amounts of missing data, again inevitable in a registry based study. The fact that all co-morbidity data was missing for patients with no public hospital stays was particularly limiting, although sensitivity analysis showed that imputed values for these patients gave almost identical coefficients. The use of public/private inpatient status as a proxy for health insurance status may also be a weakness, but given the high costs of inpatient private care it is unlikely that many patients would have opted to pay these from their own resources.

These findings could be strengthened by more focussed patient-centred research [23,62–65] to identify the pathways leading to emergency and non-emergency presentation, and their consequences for treatment and outcomes; to gain better insights into the processes of shared decision-making on treatment and how these are affected by age, co-morbidity and socio-economic status; and to develop more sensitive measures of comorbidity and fitness for treatment.
